# Factors Associated with Transition from Community Settings to Hospital as Place of Death for Adults Aged 75 and Older: A Population‐Based Mortality Follow‐Back Survey

**DOI:** 10.1111/jgs.14442

**Published:** 2016-09-09

**Authors:** Anna E. Bone, Wei Gao, Barbara Gomes, Katherine E. Sleeman, Matthew Maddocks, Juliet Wright, Deokhee Yi, Irene J. Higginson, Catherine J. Evans, OPTCare Elderly, Myfanwy Morgan, Paul McCrone, Sue Hall, Emma Gordon, Fiona Lindsay, Carla Bruni, Shamim Taherzadeh, Richard Harding, Helen Harris, Anita Wright, Sue Guerrier, John Barry, Lesley Talmey, Colin Vincent, Mike Bojczuk, Jack Hazelgrove, Rowena Vohora, Katie Stone, Mark Philips, Nina Walters, Kate Porter, Claire Cox

**Affiliations:** ^1^ Department of Palliative Care, Policy and RehabilitationCicely Saunders InstituteKing's College LondonLondonUK; ^2^ Sussex Community National Health Service Foundation TrustBrighton and HoveUK; ^3^ Brighton and Sussex Medical SchoolUniversity of SussexBrightonUK

**Keywords:** frail elderly, palliative care, terminal care, cross‐sectional survey, mortality follow‐back survey

## Abstract

**Objectives:**

To identify factors associated with end‐of‐life (EoL) transition from usual place of care to the hospital as place of death for people aged 75 and older.

**Design:**

Population‐based mortality follow‐back survey.

**Setting:**

Deaths over 6 months in 2012 in two unitary authorities in England covering 800 square miles with more than 1 million residents.

**Participants:**

A random sample of people aged 75 and older who died in a care home or hospital and all those who died at home or in a hospice unit (N = 882). Cases were identified from death registrations. The person who registered the death (a relative for 98.9%) completed the survey.

**Measurements:**

The main outcome was EoL transition to the hospital as place of death versus no EoL transition to the hospital. Multivariable modified Poisson regression was used to examine factors (illness, demographic, environmental) related to EoL transition to the hospital.

**Results:**

Four hundred forty‐three (50.2%) individuals responded, describing the care of the people who died. Most died from nonmalignant conditions (76.3%) at a mean age of 87.4 ± 6.4. One hundred forty‐six (32.3%) transitioned to the hospital and died there. Transition was more likely for individuals with respiratory disease than for those with cancer (prevalence ratio (PR) = 2.07, 95% confidence interval (CI) = 1.42–3.01) and for people with severe breathlessness (PR = 1.96, 95% CI = 1.12–3.43). Transition was less likely if EoL preferences had been discussed with a healthcare professional (PR = 0.60, 95% CI = 0.42–0.88) and when there was a key healthcare professional (PR = 0.74, 95% CI = 0.58–0.95).

**Conclusion:**

To reduce EoL transition to the hospital for older people, there needs to be improved management of breathlessness in the community and better access to a key healthcare professional skilled in coordinating care, communication, facilitating complex discussions, and in planning for future care.

People aged 75 and older are a rapidly growing demographic worldwide,^1^ accounting for approximately two‐thirds of deaths in more‐developed countries.^2^ As people are dying at increasingly older ages, patterns of diseases and causes of death are changing.^3^ Older people commonly live with multimorbidities and frailty and die from a combination of conditions.^4^ It is imperative to understand how end‐of‐life (EoL) care is delivered to this expanding and complex population group to inform health policy and optimize service provision.

Most older people die in the hospital in developed countries^5^, ^6^ despite knowledge that the majority wish to remain at home or their usual place of care at the EoL.^7^ Care homes are increasingly the usual place of care for older adults.^8^ Older people's overall preference is to remain in an environment that is safe and secure with autonomy preserved and their loved ones nearby.^9^ Transition from usual place of care at the EoL is often challenging for older adults and their families. Transition disrupts the continuity of care at the EoL and threatens the quality of care received.^10^, ^11^ Safety is also compromised, with hospitalization for older people associated with physical and cognitive decline and greater risk of mortality.^12^

A major cost driver in the provision of EoL care is inpatient hospital stay.^13^ In most high‐income countries, there is an overreliance on acute hospital care at the EoL.^13^ In the United Kingdom, there has been a reduction in hospital deaths, which may be attributed to policy initiatives to increase home‐based care at the EoL,^14^ including specialist palliative care services and home hospice (receipt of which does not affect eligibility to hospital care in the United Kingdom), but improvements have been seen mainly for those dying from cancer.^15^

Studies have explored transitions between care settings in the months before death,^10^, ^16^ place of death using national death registration data,^3^, ^18^ and outcomes of care according to place of death,^19^ but such studies rarely consider self‐ or caregiver‐reported symptoms and concerns or preferences for care and their effect on transition at the EoL to place of death. This study aimed to identify explanatory illness and individual and environmental factors in the last 3 months of life associated with transition from community settings to the hospital as place of death for people aged 75 and older.

## Methods

The study used a population‐based mortality follow‐back survey design.^20^

### Ethics Statement

A National Health Service research ethics committee (REC no. 12/LO/1367) approved the study. The Office for National Statistics (ONS) approved individual researcher access to anonymized national death registration data. Return of a completed questionnaire was taken as consent. All data were anonymized and stored securely.

### Setting

The study included two contrasting geographical areas in southern England (rural vs city) with a total population of more than 1 million and a geographical area of 800 square miles.^17^

### Sample

The sampling frame was identified from ONS death registration data and comprised people aged 75 and older who had died at home or in a care home, hospital, or inpatient hospice unit. Individuals who had died from cancer or a nonmalignant illness in the two study areas were included. All underlying causes of death common in advanced age and suitable for palliative care^21^ were selected, excluding causes of death unlikely to be suitable for palliative care (e.g., accidental deaths). Informants were those who registered the deaths (a relative in 98.9% of cases). Individuals involved in a national postbereavement survey,^22^ cases in which officials registered the death (e.g., a solicitor), and individuals with no contact address were also excluded.

Based on a sample size calculation, it was planned to include 310 deaths in the study. Standardized differences between death in the hospital and in community settings were estimated from findings on home deaths and deaths elsewhere (hospital, care home, inpatient hospice) for individuals with cancer—the best data available to inform the calculation.^23^, ^24^ Three variables were examined: preference for death at home (yes vs no; standardized difference = 0.866), help of community nurse (yes vs no; standardized difference = 0.795), and satisfaction with general practitioner care (poor vs fair, good, excellent; standardized difference = 0.225). Using 80% power and significance of .05, sample sizes needed were estimated to be 22, 26, and 310, respectively. The goal was to achieve the largest sample size estimate (310) to ensure detection of difference for each considered variable. Because of the older age of respondents, a lower response rate (35%) and higher missing data (30%) were anticipated than with similar follow‐back surveys, so to obtain the 310 participnants needed, it was planned to approach 882 people. The sample was stratified according to geographical area in the study site (rural vs city) and place of death. For the city area, all eligible deaths in each care setting were included, but for the rural area, all home and inpatient hospice deaths and a random sample of hospital (45%) and care home deaths (44%) were included to account for their high frequency.^20^

### Procedures

The ONS invited the person who registered the death to participate 4 to 10 months after death registration. The ONS mailed the QUALYCARE survey^20^ to eligible participants as a single wave in October 2012, and reminders were mailed 3 weeks (letter) and 6 weeks (letter and survey) later. A survey helpline was provided to support participants, and a research nurse was available for participants requiring face‐to‐face assistance (e.g., because of visual impairment).

The QUALYCARE survey is an adapted short form of the Cartwright survey developed in the 1960s to measure bereaved relatives' perspectives of their loved ones' experiences in the last year of life.^25^ The survey includes validated measures of palliative symptoms and problems experienced in the last week of life (Palliative care Outcome Scale, POS)^26^ and of health and social care services use and informal care in the last 3 months of life (Client Service Receipt Inventory).^27^ It also asks about the decedent's preferences for place of death (as far as the respondent knows), the respondent's preferences (looking back 3 months before death), and whether preferences had been discussed. The original QUALYCARE survey was designed for adults with cancer.^20^, ^28^ It was modified to customize it for older people with cancer or nonmalignant conditions. The study's Steering Group and Lay Project Advisory Group oversaw this process. The ONS provided data on cause of death, contributing causes of death, place of death, age, and a national composite measure of area deprivation (Indices of Multiple Deprivation) using decedents' usual residence at the Lower Super Output Area and analyzed in quintiles.^29^

### Main Outcome

The main outcome was transition to the hospital as the place of death versus no EoL transition to the hospital. The informant identified usual place of care, which was defined as the place where the decedent spent most of his or her last 3 months of life. Decedents who had usual care at home or a friend's or relative's own home constituted the “at home” group, and those with nursing care (nursing home) or with personal care only (residential care) constituted the “care home” group.

### Explanatory Variables

Factors associated with EoL transition to hospital as place of death were examined, with explanatory variables selected based on previous research and clinical judgement.^3^, ^18^, ^30^ An explanatory model^30^ was used to categorize variables as illness factors (underlying cause of death, symptom distress (e.g., pain), psychological distress (e.g., anxiety), caregiver anxiety), individual factors (e.g., age, sex), and environmental factors at the individual level (e.g., healthcare input). Underlying cause of death was grouped into *International Classification of Diseases, Tenth Revision* (ICD‐10) top‐level disease codes (e.g., respiratory ICD‐10 J). Frailty was defined using ICD‐10 R54 (senility) and collapsed with Alzheimer's disease (ICS10‐F01, F03), dementia (ICD‐10 G30), and other causes of death. A count of contributing causes of death was used (including underlying cause of death).

POS data on decedents' symptoms and concerns in the last week of life were used, with five response options ranging from not at all to overwhelmingly and missing data imputed using median values. Service use (Client Service Receipt Inventory) was analyzed as a continuous variable for frequently used services (e.g., general practitioner) and dichotomous yes or no for specialist services. Missing data were imputed using the lower quartile number of contacts that showed a positively skewed distribution. Missing data (17.5%) were imputed for the variable “Do you feel he/she had a key contact person (healthcare professional) to rely on to get things done?” using a proxy variable of contact with a specialist palliative care team or a specialist nurse (e.g., respiratory nurse) because these groups commonly perform a key worker role^31^, ^32^ and explored with sensitivity analyses.

### Data Analysis

To compare EoL transition to the hospital as place of death (defined as 1) with no EoL transition or transition to a care home or inpatient hospice unit (defined as 0), univariate associations with illness‐related, individual, and environmental factors were determined using the chi‐square test or Mann–Whitney test as appropriate.

Multivariable modified Poisson regression with robust error variance was used to estimate prevalence ratios (PRs) for EoL transition to the hospital.^33^ Modified Poisson regression was favored over logistic regression because a PR was considered a preferable measure of risk than an odds ratio, which may overestimate effect size.^33^ A propensity score (probability of participation) was generated using factors significantly associated with participation (age and place of death; Table S1). The inverse propensity score was used in the regression model to adjust for response bias.^34^ Findings from the univariate analysis and clinical consideration informed candidate variables for regression modeling (Table S2). Collinearity between explanatory variables was assessed to inform variable inclusion using Spearman rank correlation coefficients and the chi‐square test as appropriate. Backward selection was used to determine which variables were entered into the final parsimonious model. Age, sex, and place of usual care were forced to remain as potential confounders. Sensitivity analyses of the dependent variable (Table S3) and variables that were significant in the univariate analysis (e.g., specialist palliative care variable (Table S4) were used to explore case inclusion. Analyses were conducted using Stata SE version 13 (Stata Corp., College Station, TX).

## Results

### The Sample

#### Informants

Four hundred forty‐three informants completed the survey (50.2% response rate); 438 (98.9%) were related to the decedent, mostly a child (n = 303, 68.4%) or a spouse or partner (n = 68, 15.8%). Mean age was 62.3 ± 10.7, 279 (63.0%) were women, 195 (44.0%) were in paid employment, and 170 (38.4%) were retired. Two hundred twenty‐three (50.3%) were from area 1 (rural) and 220 (49.7%) from area 2 (city).

#### Decedents

Decedent age ranged from 75 to 104 (mean 87.4 ± 6.4) (Table 1). Two hundred sixty‐two were women (59.1%), 237 were widowed (53.5%), and 410 were white (92.5%). The main underlying causes of death were circulatory diseases (n = 144, 32.5%), respiratory conditions (n = 90, 20.3%), cancer (n = 105, 23.7%), and dementia or frailty (n = 88, 19.9%) (Table 1); 39.7% of decedents had a mention of frailty or dementia on their death certificate. Frailty was a contributing cause of death for 21.9% (n = 97) and dementia for 6.7% (n = 30).

**Table 1:** Decedent and Informant Characteristics (N = 443)

Characteristic	n (%)
Decedent's relationship to informant, n (%)	
Husband, wife, partner	68 (15.4)
Child	303 (68.4)
Other relative	67 (15.1)
Friend or staff (e.g., care home)	5 (1.1)
Decedent sex, n (%)	
Male	181 (40.9)
Female	262 (59.1)
Decedent age	
Mean ± standard deviation	87.4 ± 6.4
75–79, n (%)	51 (11.5)
80–84, n (%)	103 (23.3)
85–89, n (%)	111 (25.1)
90–94, n (%)	117 (26.4)
≤95, n (%)	61 (13.8)
Decedent ethnicity, n (%)	
White British	410 (92.6)
White other, other ethnicity	18 (4.1)
Quintile of deprivation of decedent's area of residence (Indices of Multiple Deprivation), n (%)	
1 (most deprived)	51 (11.5)
2	69 (15.6)
3	111 (25.1)
4	90 (20.4)
5 (least deprived)	121 (27.4)
Underlying cause of death (*International Classification of Diseases Tenth Revision*, code), n (%)	
Cancer (C)	105 (23.7)
Ischemic heart disease (I20–125)	69 (15.6)
Other circulatory (I [other])	52 (11.7)
Cerebrovascular (I60–169)	23 (5.2)
Respiratory (J)	90 (20.3)
Dementia (F01‐F03, G30)	67 (15.1)
Frailty (R54)	21 (4.7)
Other	16 (3.6)
Place of death, n (%)	
Home^a^	120 (27.1)
Care home^b^	138 (31.2)
Inpatient hospice	23 (5.2)
Hospital	162 (36.6)
Usual place of care in last 3 months of life, n (%)	
Home^a^	254 (57.3)
Care home^b^	160 (36.1)
Inpatient hospice	2 (0.5)
Hospital	27 (6.1)
Decedent's preferred place of death (as far as respondent knew), n (%)	
Home^a^	301 (68.0)
Care home^b^	42 (9.5)
Inpatient hospice	14 (3.2)
Hospital	9 (2.0)
≥2 preferences	5 (1.1)
No preference	21 (4.7)
Don't know	37 (8.3)5
Informant's preferred place of death (3 months before death), n (%)	
Home^a^	186 (42.0)
Care home^b^	111 (25.1)
Inpatient hospice	27 (6.1)
Hospital	43 (9.7)
No preference	58 (13.1)
≥2 preferences	7 (1.6)
End‐of‐life transition, n (%)	
To hospital	146 (33.0)
To community institution	
Care home^b^	20 (4.5)
Inpatient hospice	21 (4.7)
No transition	
Home^a^	120 (27.1)
Care home^b^	118 (26.6)
Inpatient hospice	2 (0.5)
Hospital	16 (3.6)

Some column percentages do not sum to 100% because of missing data.

a Own home or home of friend or relative.

b Nursing or residential home.

### Transition to the Hospital at the EoL

One‐third (32.3%) of the sample transitioned to the hospital at the EoL as their place of death. Only 2% of respondents expressed a known preference of the individual to die in the hospital, and 9.7% of respondents expressed a preference for the decedent to have died in the hospital. Most people wished to die at home (67.9%), and family members, looking back at the 3 months before death, expressed a lower preference for home (42%) but higher for care home (25.1%, vs 9.5% for decedents) (Table 1).

**Figure 1:**
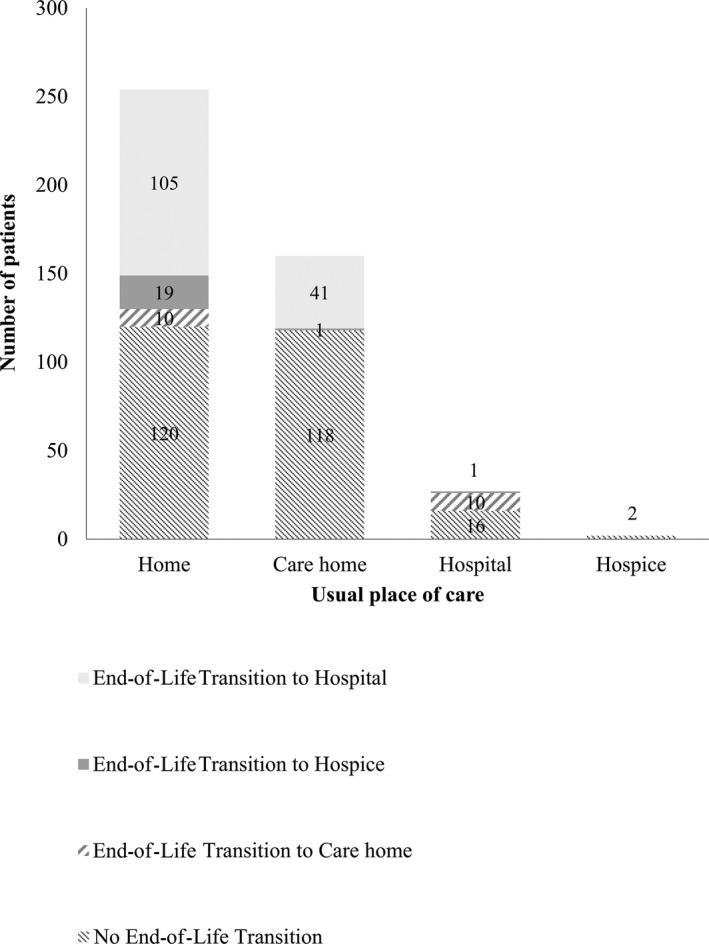
Final transition to place of death by usual place of care in the last 3 months of life.

The majority of the decedents who transitioned at the EoL to the hospital as place of death did so from home (71.9%), and the remainder transitioned from a care home (28.1%) (Figure 1). Nearly half transitioned to hospital 1 week to 1 month before death (47.3%). One‐quarter (26.7%) moved in the last week of life and 10.3% in the last 24 hours of life; 14.4% spent 1 to 6 months in the hospital before death.

Two hundred forty decedents (54.2%) remained in their usual place of care in a community setting—mainly home (n = 120, 27.1%) or care home (n = 118, 26.6%). A small number transitioned in the last weeks of life to an inpatient hospice unit (n = 21, 4.7%) or a care home (n = 20, 4.5%). For 16 (3,6%), usual place of care in the last 3 months and place of death was a hospital (Figure 1). These cases were considered anomalies and were thus excluded from the analysis.

### Adjusted Associations with EoL Transition to the Hospital

EoL transition to the hospital was associated with factors relating to the individual's illness and symptom distress, caregiver anxiety, and type and volume of healthcare received (Table S2). Seven variables were included in the final multivariable model of factors related to EoL transition to the hospital (Table 2). These included factors related to the person's illness and receipt of health care. Dying from respiratory or circulatory diseases was independently associated with EoL transition to the hospital. Individuals dying from a respiratory disease were twice as likely to transition to the hospital at the EoL (PR = 2.07, 95% confidence interval (CI) = 1.42–3.01), and those dying from circulatory disease were 53% more likely to transition to the hospital at the EoL (PR = 1.53, 95% CI = 1.06–2.20) than those dying from cancer. Individuals experiencing severe to overwhelming breathlessness in their last week of life were almost twice as likely to transition to the hospital at the EoL as those who experienced no breathlessness (PR = 1.96, 95% CI = 1.12–3.43).

**Table 2:** Multivariable Regression Analysis: End‐of‐Life (EoL) Transition to Hospital Versus No Transition or Transition to Community Setting

Variable	PR (95% Confidence Interval)	*P*‐Value
Age	1.00 (0.98–1.02)	.90
Female	1.21 (0.94–1.55)	.13
Usual place of care, home^a^	1.80 (1.35–2.40)	<.001
Cause of death (reference cancer)		
Circulatory disease	1.53 (1.06–2.20)	.02
Respiratory disease	2.07 (1.42–3.01)	<.001
Other including frailty and dementia	1.01 (0.60–1.70)	.98
Breathlessness (reference not at all)		
Slightly to moderately	1.46 (0.83–2.56)	.19
Severely to overwhelmingly	1.96 (1.12–3.43)	.02
Discussed preferred place of care with health professional	0.60 (0.42–0.88)	.008
Key health professional	0.74 (0.58–0.95)	.02

N = 424 (3 excluded for missing data on key health professional variable). Regression model is weighted by the inverse propensity score. A prevalence ratio (PR) >1 indicates higher probability of EoL transition to hospital.

a Other settings include care home (n = 160), inpatient hospice (n = 2), hospital with transition (n = 11) (Figure 1).

Individuals who discussed their preferred place of care with a healthcare professional were less likely to transition to the hospital (PR = 0.60, 95% CI = 0.42–0.88). Similarly, individuals who had a key healthcare professional to rely on to get things done were less likely to transition to the hospital (PR = 0.74, 95% CI = 0.58–0.95). Sensitivity analyses to explore the effect of imputed values for included variables (complete cases) demonstrated consistent findings. Specialist palliative care was not significantly associated with transition to the hospital and was excluded from the model (OR = 0.79, 95% CI = 0.56–1.13) (Table S4). This finding may be attributed to the significant associations between specialist palliative care and the covariates cause of death, presence of a key worker, and discussions about preferred place of care with a health professional (chi‐square test *P* < .001 for each).

## Discussion

This population‐based study of the EoL care of older adults found that more than one‐third transitioned to the hospital at the EoL and died there, even though only 2.0% wished to die in this setting. There is an apparent reliance on hospitals to provide EoL care for older people, particularly those living at home, who accounted for 71.9% of those who transitioned. Very few (4.7%) older people transitioned to an inpatient hospice unit at the EoL. Almost half (47.3%) of the older people who transitioned to the hospital were inpatients for several weeks to a month before death. Irrespective of their usual place of care, the likelihood of transition to the hospital was greater for people with respiratory and circulatory disease and with severe breathlessness. The likelihood of transition was lower for people who had discussed EoL care preferences with a health professional and those with an identified key healthcare professional.

Breathlessness is a prominent symptom of individuals with various advanced illnesses^35^ and is a common symptom in individuals in the emergency department.^36^ Severe breathlessness is distressing for individuals and their caregivers,^37^ which may explain the observed association with transition to the hospital. The current study findings suggest that there is a need to better alleviate the symptom of breathlessness for older adults at the EoL. There is growing evidence that innovative breathlessness services improve outcomes of mastery of breathlessness and distress.^38^, ^39^ Improved breathlessness services provided by specialist health professionals in the community may reduce the incidence of transition to the hospital for older people.

Transition to the hospital as place of death was more likely in people with certain nonmalignant illnesses (respiratory and circulatory diseases). Those who died from nonmalignant respiratory disease were twice as likely to transition to the hospital at the EoL as those with cancer, and the association remained after adjusting for breathlessness. Studies consistently report the association between respiratory disease and dying in the hospital.^3^, ^40^, ^41^ For individuals with respiratory disease such as chronic obstructive pulmonary disease, EoL is often hard to recognize and inadequately anticipated.^42^ This may preclude timely access to palliative care interventions and services. With prognostic uncertainty, indicators of unstable or deteriorating symptoms and concerns, notably unplanned hospital attendance, may better indicate requirement for palliative interventions.^42^ Most older people who died in the hospital had been admitted several weeks before death, suggesting that there was opportunity for anticipation of EoL, palliative care input, and discussions about wishes for future care.

Discussion of preferences for future care with a health professional and presence of a key worker—a healthcare professional to rely on to get things done—were protective factors against transition to the hospital. A key worker with clinical expertise may augment continuity of care through better care coordination and timely access to services.^31^, ^43^ Continuity is a central component of quality of health care, comprising relational continuity between a person and a clinician and management continuity between clinicians (e.g., information sharing).^44^ Relational continuity with a key healthcare professional may facilitate discussions with individuals and families on preferences for future care. Few people (22.3%) were reported to have discussioned preferences for future care with a health professional. These findings suggest that EoL discussions and wider provision of advance care planning may be important in enabling people to remain in their usual place of care at the EoL or to shorten the length of hospital stay.

It is clinically challenging to manage older people nearing the EoL because they often have multiple debilitating conditions with complex needs, including physical, emotional, psychological, and spiritual.^45^ It is important that a key worker have the clinical skills and training to assess and anticipate care needs effectively, coordinate timely care, and discuss sensitively preferences for future care at the EoL with older people and their families.^46^ Older adults with multiple nonmalignant conditions have palliative care needs similar to those of individuals with advanced cancer.^35^, ^45^ Home palliative care services have been effective for people with cancer in improving symptom management, including alleviation of breathlessness, and in more than doubling the incidence of home deaths.^47^ There is a need for similar service and treatment innovation for older adults living in the community and for evaluation using robust trials to provide high‐quality evidence of potential effect.

The study's strengths are the focus on people aged 75 and older in a range of care settings, including care homes, and with multiple conditions and the high response rate (50.2%).^22^, ^48^ Few studies have examined in detail the factors associated with EoL transition to a hospital for older adults.^49^ The linkage of national death registration data with bereaved relatives' accounts has enabled detailed and unique reporting.

A limitation of the study is the use of a mortality follow‐back survey reliant on proxy accounts, although the majority of proxy informants were close family members, mainly sons or daughters. Although expected, the nonresponse rate and ethnic homogeneity of the sample limit the generalizability of the findings. To minimize the effect of systematic nonresponse on the findings, known differences in decedent age and place of death between participants and nonparticipants were accounted for in the analyses. Home and inpatient hospice deaths were oversampled, including all cases to enable analysis of these less‐common places of death. The proportion that transitioned to the hospital at EoL is therefore likely to be underestimated. Appropriate and inappropriate hospital admission at the EoL were not differentiated in the analysis.

In conclusion, these findings suggest that reducing reliance on hospital care at the EoL for older people with nonmalignant conditions requires timely and coordinated services responsive to increasing symptom distress and greater anticipatory care planning. Policy imperatives are improved management of breathlessness in the community and greater emphasis on an assigned key healthcare professional skilled in coordinating care, communication, facilitating complex discussions, and future care planning. Further research is required on evaluating service innovations to improve provision of palliative and EoL care and access for older people with nonmalignant conditions in community settings.

## Supporting information


**Table S1.** Comparison of decedent characteristics for participants and nonparticipants.
**Table S2.** Unadjusted univariate analysis of illness and environmental factors by EoL transition.
**Table S3.** Sensitivity analysis on dependent variable.
**Table S4.** Sensitivity analysis for multivariable regression of EoL transition to hospital versus no transition or transition to community setting.
