# Supplementary material for: Factors Associated with Transition from Community Settings to Hospital as Place of Death for Adults Aged 75 and Older: A Population‐Based Mortality Follow‐Back Survey
Source: J Am Geriatr Soc. 2016 Sep 9;64(11):2210–7. doi: 10.1111/jgs.14442 (PMC5324592; doi:10.1111/jgs.14442)
Supplement: Supplementary file 1 — Table S1. Comparison of decedent characteristics for participants and nonparticipants. Table S2. Unadjusted univariate analysis of illness and environmental factors by EoL transition. Table S3. Sensitivity analysis on dependent variable. Table S4. Sensitivity analysis for multivariable regression of EoL transition to hospital versus no transition or transition to community setting. [file JGS-64-2210-s001.docx]

SUPPLEMENTARY MATERIAL FOR ONLINE ONLY

Supplementary Table 1: Comparison of Characteristics of Participants and Nonparticipants

| Characteristic | Nonparticipants, n=439 | Participants, n=443 | P-Value |
| --- | --- | --- | --- |
| Sex |  |  | 2.70^a^ |
| Male | 185 (42.1) | 181 (41.1) |  |
| Female | 254 (57.9) | 262 (59.1) |  |
| Age |  |  |  |
| Median (interquartile range) | 86 (10) | 88 (9) | .001^b^ |
| Mean±standard deviation | 86.1±6.5 | 87.4±6.4 |  |
| 75–79, n (%) | 84 (19.1) | 51 (11.5) | .01^a^ |
| 80–84, n (%) | 107 (24.4) | 103 (23.3) |  |
| 85–89, n (%) | 107 (24.4) | 111 (25.1) |  |
| 90–94, n (%) | 94 (21.4) | 117 (26.4) |  |
| ≥95, n (%) | 47 (10.7) | 61 (13.8) |  |
| Cause of death, n (%) |  |  | .28^a^ |
| Cancer | 125 (28.5) | 105 (23.7) |  |
| Ischemic heart disease | 54 (12.3) | 69 (15.6) |  |
| Cerebrovascular | 27 (6.2) | 23 (5.2) |  |
| Other circulatory | 63 (14.4) | 52 (11.7) |  |
| Respiratory | 70 (15.9) | 90 (20.3) |  |
| Dementia | 61 (13.9) | 67 (15.1) |  |
| Frailty | 18 (4.1) | 21 (4.7) |  |
| Other | 21 (4.8) | 16 (3.6) |  |
| Place of death, n (%) |  |  | .005^a^ |
| Home | 84 (19.1) | 120 (27.1) |  |
| Care home | 124 (28.2) | 138 (31.2) |  |
| Inpatient hospice | 36 (8.2) | 23 (5.2) |  |
| Hospital | 195 (44.4) | 162 (36.6) |  |
| Indices of Multiple Deprivation^c^ |  |  | .09^a^ |
| 1 (most deprived) | 54 (12.3) | 51 (11.5) |  |
| 2 | 98 (22.3) | 69 (15.6) |  |
| 3 | 96 (21.9) | 111 (25.1) |  |
| 4 | 73 (16.6) | 90 (20.4) |  |
| 5 (least deprived) | 118 (26.9) | 121 (27.4) |  |

^a^Chi-square test.

^b^Mann-Whitney test.

^c^Missing value for 1 participant.

Supplementary Table 2: Unadjusted Univariate Analysis of Illness and Environmental Factors According to End-of-Live (EoL) Transition (N=427)

| Variable | EoL Transition to Hospital, n=146 | No EoL Transition or Transition to Inpatient Hospice or Care Home, n=281 | P-Value | Missing, n (%) |
| --- | --- | --- | --- | --- |
| Illness, n (%) |  |  |  |  |
| Cause of death |  |  | <.001^a^ |  |
| Cancer | 25 (17.1) | 77 (27.4) |  |  |
| Circulatory disease | 53 (36.3) | 83 (29.5) |  |  |
| Respiratory disease | 48 (32.9) | 37 (13.2) |  |  |
| Other including dementia and frailty | 20 (13.7) | 84 (29.9) |  |  |
| Number of mentioned causes of death |  |  |  |  |
| 1 | 17 (11.6) | 74 (26.3) | <.001^a^ |  |
| 2 | 30 (20.6) | 107 (38.1) |  |  |
| 3 | 28 (19.2) | 54 (19.2) |  |  |
| ≥4 | 71 (48.6) | 46 (16.4) |  |  |
| Pain |  |  | .27^a^ | 48 (11.2) |
| None | 52 (41.6) | 103 (40.6) |  |  |
| Slight to moderate | 62 (49.6) | 114 (44.9) |  |  |
| Severe to overwhelming | 11 (8.8) | 37 (14.6) |  |  |
| Breathlessness |  |  | <.001^a^ | 38 (8.9) |
| None | 10 (7.3) | 44 (17.5) |  |  |
| Slight to moderate | 51 (37.0) | 120 (47.8) |  |  |
| Severe to overwhelming | 77 (55.8) | 87 (34.7) |  |  |
| Depression |  |  | .77^a^ | 44 10.3) |
| None | 51 (39.2) | 99 (39.1) |  |  |
| Occasionally, sometimes | 47 (36.2) | 99 (39.1) |  |  |
| Most of the time, definitely | 32 (24.6) | 55 (21.7) |  |  |
| Anxiety |  |  | .55^a^ | 27 (6.3) |
| None | 39 (28.1) | 87 (33.3) |  |  |
| Occasionally, sometimes | 77 (55.4) | 133 (51.0) |  |  |
| Most of the time, thought of nothing else | 23 (16.6) | 41 (15.7) |  |  |
| Family anxiety |  |  | .02^a^ | 8 (1.9) |
| None | 11 (7.6) | 37 (13.5) |  |  |
| Occasionally, sometimes | 31 (21.5) | 81 (29.5) |  |  |
| Most of the time, always | 102 (70.8) | 157 (57.1) |  |  |
| Felt at peace |  |  | .27^a^ | 33(7.7) |
| All or most of the time | 64 (49.6) | 154 (58.1) |  |  |
| Some of the time, occasionally | 31 (24.0) | 51 (19.3) |  |  |
| Not very often, not at all | 34 (26.4) | 60 (22.6) |  |  |
| Environmental |  |  |  |  |
| Usual place of care, n (%) |  |  | ^c^ |  |
| Home | 105 (71.9) | 149 (53.0) |  |  |
| Care home | 41 (28.1) | 119 (42.4) |  |  |
| Inpatient hospice | 0 (0.0) | 2 (0.7) |  |  |
| Hospital | 0 (0.0) | 11 (3.9) |  |  |
| Discussed preferred place of care with clinician, n (%) |  |  | .002^a^ | 8 (1.9) |
| No, don’t know | 123 (85.4) | 197 (71.6) |  |  |
| Yes | 21 (14.6) | 78 (28.4) |  |  |
| Key healthcare professional, n (%)^d^ |  |  | <.001^a^ | 3 (0.7) |
| No | 85 (58.6) | 110 (39.4) |  |  |
| Yes | 60 (41.4) | 169 (60.6) |  |  |
| Number of face-to-face contacts with general practitioner, mean±SD | 3.6±4.6 | 4.4±4.5 | .002^b^ | 2 (0.5) |
| Number of community nurse contacts, mean±SD | 3.8±9.9 | 4.7±12.4 | .80^b^ | 9 (2.1) |
| Specialist palliative care, n (%)^e^ |  |  |  |  |
| No, don’t know | 113 (77.4) | 188 (66.9) | .02^a^ |  |
| Yes | 33 (22.6) | 93 (33.1) |  |  |

Symptoms relate to last week of life and health service to the last 3 months of life.

P-values from ^a^chi-square test of association, ^b^Mann-Whitney test.

^c^Chi-square test not performed because of 0 cell counts.

^d^Missing values for key healthcare professional (n=76, 17%) were imputed using specialist palliative care–specialist nurses variable.

^e^Specialist palliative care in all settings, including hospice care, palliative care, Marie Curie or Macmillan or any other specialist palliative care service.

SD= standard deviation.

Supplementary Table 3: Sensitivity Analysis for Dependent Variable

| Variable | Model 1 (N=424) | Model 2 (N=413) | Model 3 (N=383) |
| --- | --- | --- | --- |
|  | Prevalence Ratio (95% Confidence Interval) P-Value | | |
| Age | 1.00 (0.98–1.02) .90 | 1.00 (0.98–1.02) .87 | 1.00 (0.98–1.02) .79 |
| Female | 1.21 (0.94–1.55) .13 | 1.18 (0.92–1.51) .18 | 1.22 (0.97–1.54) .09 |
| Usual place of care: home | 1.80 (1.35–2.40) <.001 | 1.66 (1.24–2.21) .001 | 1.78 (1.34–2.36) <.001 |
| Cause of death (reference cancer) |  |  |  |
| Circulatory disease | 1.53 (1.06–2.20) .02 | 1.45 (1.01–2.09) .046 | 1.15 (0.84–1.57) .37 |
| Respiratory disease | 2.07 (1.42–3.01) <.001 | 1.99 (1.37–2.89) <.001 | 1.53 (1.11–2.10) .01 |
| Other, including frailty and dementia | 1.01 (0.60–1.70) .98 | 0.92 (0.54–1.57) .77 | 0.73 (0.45–1.20) .22 |
| Breathlessness (reference none) |  |  |  |
| Slight, moderate | 1.46 (0.83–2.56) .19 | 1.44 (0.82–2.53) .20 | 1.58 (0.91–2.77) .11 |
| Severe, overwhelming | 1.96 (1.12–3.43) .02 | 1.93 (1.11–3.38) .02 | 2.12 (1.22–3.70) .008 |
| Discussed preferred place of death with health professional | 0.60 (0.42–0.88) .008 | 0.60 (0.42–0.87) .007 | 0.64 (0.45–0.90) .01 |
| Key health professional | 0.74 (0.58–0.95) .02 | 0.73 (0.57–0.93) .01 | 0.77 (0.61–0.97) .02 |

Model 1: Final model with EoL transition to hospital (1) vs no EoL transition/ transition to care home or inpatient hospice (0).

Model 2: Excluded EoL transition to community from hospital (n=11)

Model 3: Excluded EoL transition to community from hospital and other community settings (n=41).

Supplementary Table 4: Sensitivity Analysis for Multivariable Regression of End-of-Life (EoL) Transition to Hospital Versus No Transition or Transition to Community Setting (N=424)

| Variable | PR (95% Confidence Interval) P-Value |
| --- | --- |
| Age | 1.00 (0.98–1.02) .96 |
| Female | 1.22 (0.96–1.56) .12 |
| Usual place of care: home^a^ | 1.86 (1.34–2.50) <.001 |
| Cause of death (reference cancer) |  |
| Circulatory disease | 1.37 (0.91–2.06) .13 |
| Respiratory disease | 1.88 (1.25–2.82) .002 |
| Other including frailty and dementia | 0.93 (0.54–1.58) .78 |
| Breathlessness (reference none) |  |
| Slight, moderate | 1.45 (0.82–2.56) .19 |
| Severe, overwhelming | 1.97 (1.11–3.47) .02 |
| Discussed preferred place of care with health professional | 0.64 (0.44–0.93) .02 |
| Key health professional | 0.76 (0.59–0.97) .03 |
| Specialist palliative care^b^ | 0.79 (0.56–1.13) .19 |

Three individuals were excluded because of missing data on key health professional variable. Regression model is weighted by the inverse propensity score.

Prevalence ratio (PR)>1 indicates higher probability of EoL transition to hospital.

^a^Versus care home (n=160), inpatient hospice (n=2), hospital with transition (n=11) (Figure 1).

^b^Specialist palliative care in all settings, including hospice care, palliative care, Marie Curie, Macmillan, and any other specialist palliative care service.
